# Strategy for 90% autoverification of clinical chemistry and immunoassay test results using six sigma process improvement

**DOI:** 10.1016/j.dib.2018.04.080

**Published:** 2018-05-03

**Authors:** Edward W. Randell, Garry Short, Natasha Lee, Allison Beresford, Margaret Spencer, Marina Kennell, Zoë Moores, David Parry

**Affiliations:** a Discipline of Laboratory Medicine, Eastern Health Authority, 300 Prince Philip Dr., St. John’s, NL, Canada A1B 3V6; b Faculty of Medicine, Memorial University of Newfoundland, 300 Prince Philip Dr., St. John’s, NL, Canada A1B 3V6; c Western Health Authority, 1 Brookfield Ave., Corner Brook, NL, Canada A2H 6J7

## Abstract

Six Sigma involves a structured process improvement strategy that places processes on a pathway to continued improvement. The data presented here summarizes a project that took three clinical laboratories from autoverification processes that allowed between about 40% to 60% of tests being auto-verified to more than 90% of tests and samples auto-verified. The project schedule, metrics and targets, a description of the previous system and detailed information on the changes made to achieve greater than 90% auto-verification is presented for this Six Sigma DMAIC (Design, Measure, Analyze, Improve, Control) process improvement project.

**Specifications Table**

**Table:** 

Subject area	*Clinical Biochemistry*
More specific subject area	*Six sigma process improvement*
Type of data	*Tables and figures*
How data was acquired	*Making use of open database connectivity downloads from Instrument Manager (Data Innovations) middleware for tests analyzed on Architect c-series and i-series instruments (Abbott) were made; Manual timed activities using a stopwatch and observer.*
Data format	*Raw and analyzed*
Experimental factors	*Not Applicable*
Experimental features	*Six sigma process improvement strategy was applied to improve laboratory test auto-verification*
Data source location	*St. Clare’s Mercy Hospital and Health Sciences Centre in St. John’s; and Western Memorial Hospital in Corner Brook, Canada.*
Data accessibility	*Raw data is maintained with the corresponding author.*

**Value of the data**

• Provides outline for Six Sigma process improvement design for auto-verification processes.
• Provides benchmarks and metrics to monitor and assess auto-verification processes.
• Describes test specific auto-verification parameters and consistency checks to achieve 90% auto-verification.
• Provides brief notes to medical laboratory technologists and basic strategies to address delta check and extreme values held for manual review.

## 1 Data

The data presented is from three clinical chemistry laboratories in Newfoundland and Labrador where Six Sigma process improvement methodology was used to improve the efficiency of autoverification (AV) processes affecting clinical chemistry and immunoassay tests. Data includes baseline data from all three laboratories (HSC-Health Science Centre; WMH-Western Memorial Hospital; and SCH-St. Clare’s Mercy Hospital), test specific parameters for the new AV system, and other tools to assist with operation of the new AV program which achieved greater than 90% sample AV at the three sites examined. The original AV system is described, specific changes made, and some effects on the changes.

## 2 Experimental design, materials and methods

**Fig. 1:**
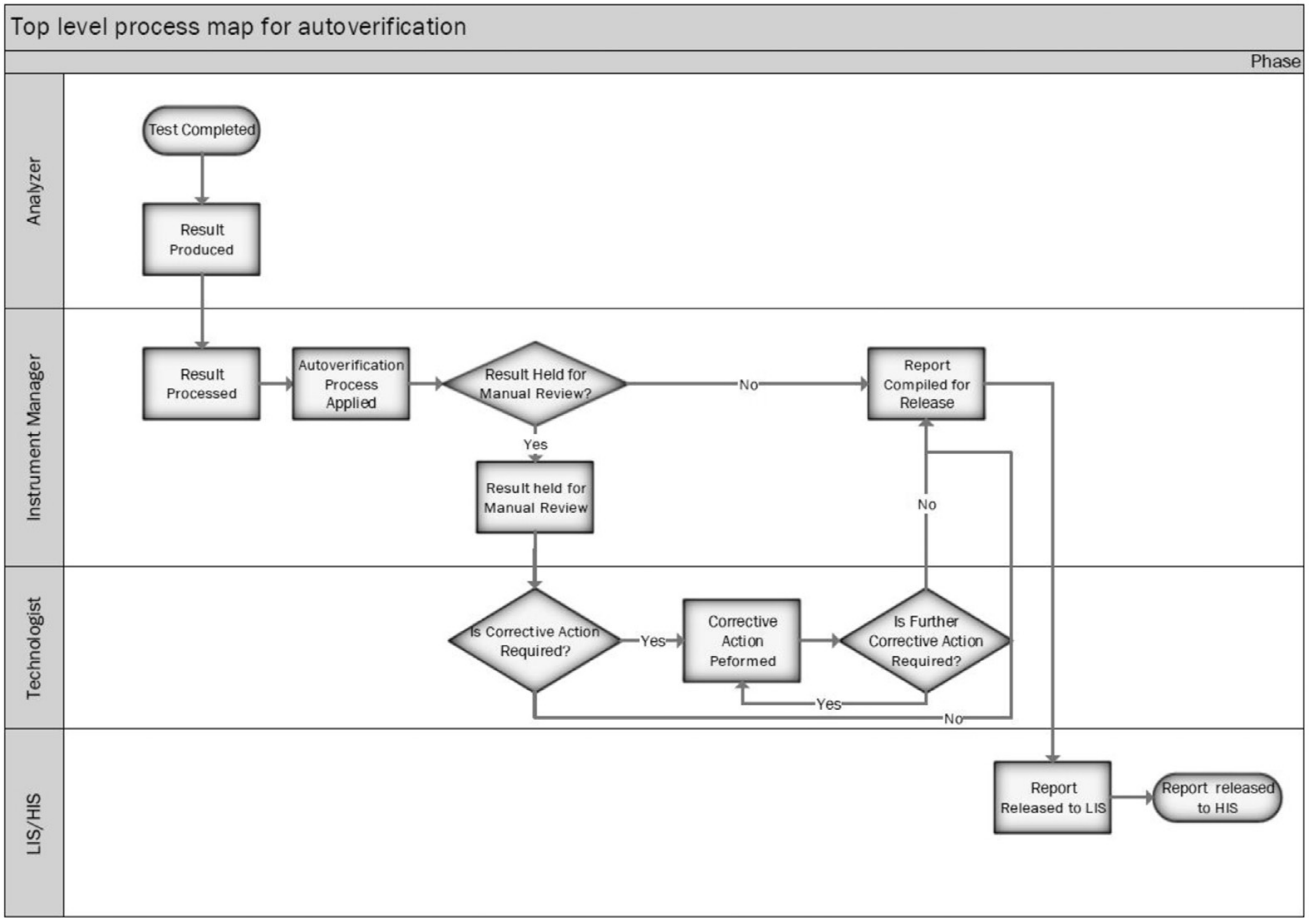
Top level process map describing the AV work flow. This swim-lane diagram identifies actions done by the automated analyzer, the middle ware software (Instrument Manager), the MLT (or technologist), and laboratory/hospital information system (LIS/HIS).

**Fig. 2:**
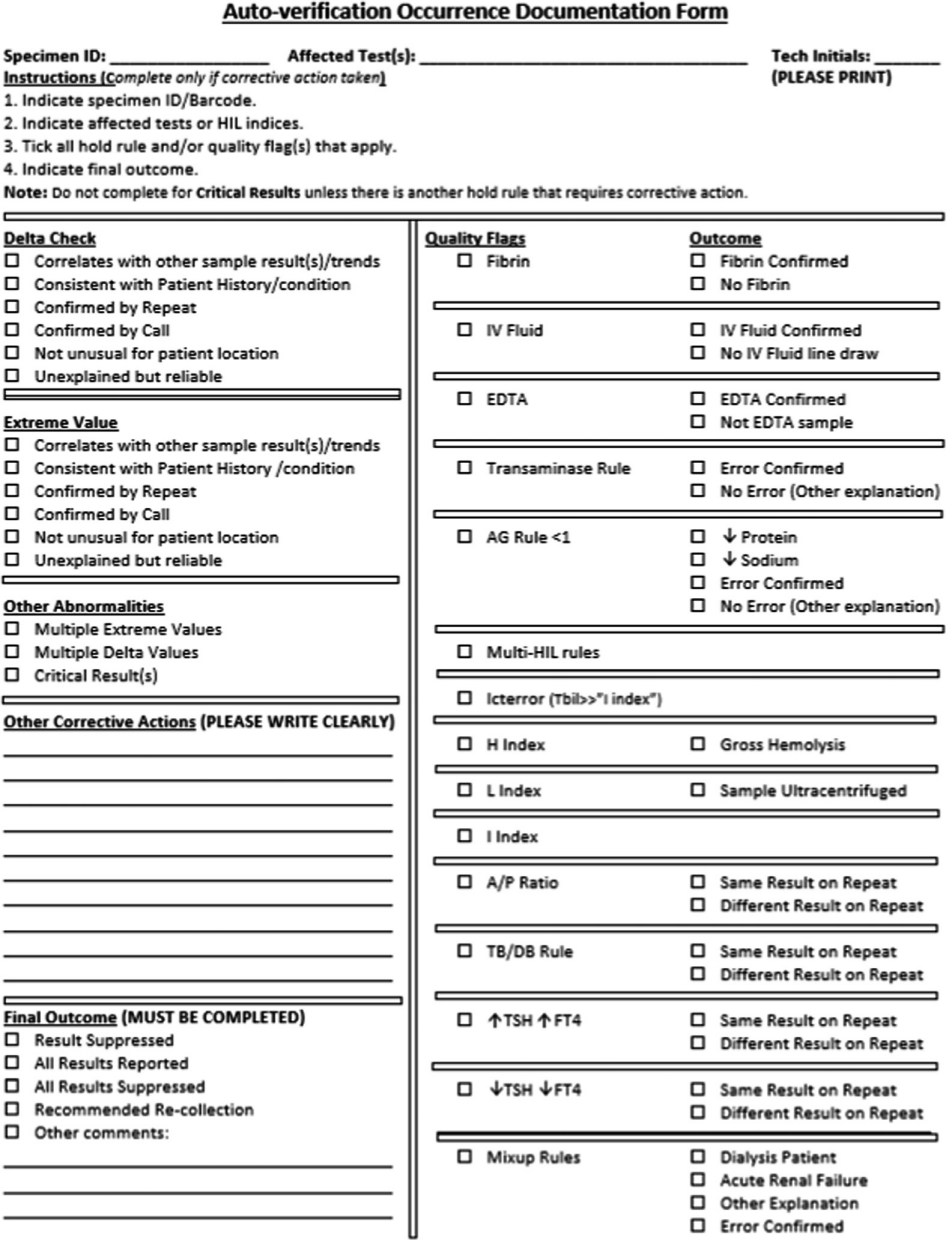
Post-improvement occurrence documentation form. Quality flags indicate consistency checks and various HIL flags.

**Fig. 3:**
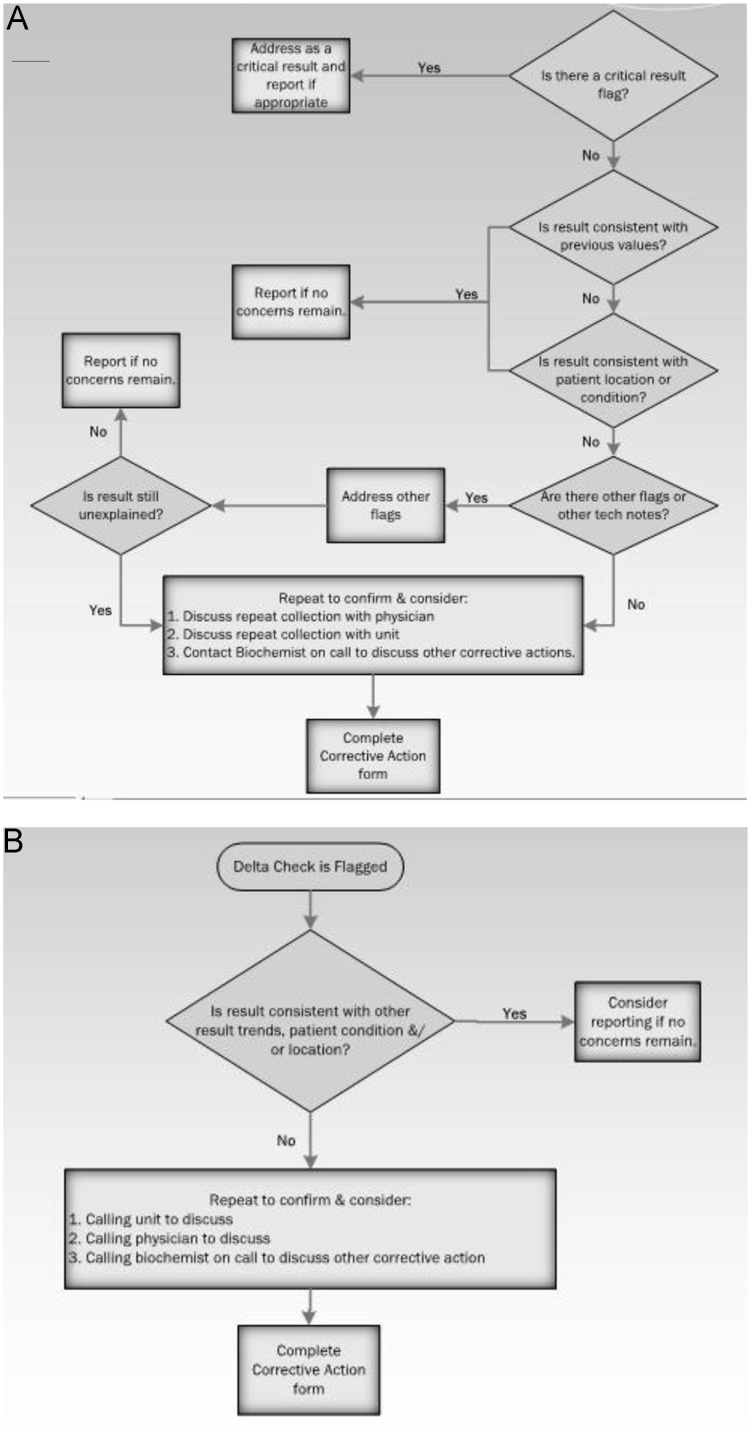
Decision tree for tests held as extreme results (A) and delta checks (B).

A Six Sigma process improvement effort carried out to improve AV processes at the three sites [1]. All sites had similar AV routines starting out. An outline of the Six Sigma process improvement schedule based on DMAIC (Design, Measure, Analyze, Improve, Control) methodology is provided in Table 1. The project team consisted of thirteen-members representing managers, clinical biochemists, front line staff and others. The process metrics and benchmarks/targets were established during the “Design and Measurement” phases. Various process maps including Fig. 1 which outlines the patient result verification workflow were also constructed to better understand the AV process. The reliability and reproducibility of all process metrics were validated and are listed in Table 2 along with baseline and benchmarks or targets for each metric. Baseline values for most metrics were mainly determined from download and analysis of test order specific information from Instrument manager (IM) middleware. An exception was test manual verification time which was determined by an observer who timed by stop watch the manual verification activities by medical laboratory technologists (MLTs) both during the Measurement Phase but also later during the Control phase. The new AV scheme (parameters detailed in Supplementary Table 3) was developed following review of process metrics and examination of the original system, and by several rounds of meetings with MLTs at the three sites in order to gain insight on manual verification activities. The key changes made and their predicted impact on test hold rates are summarized in Table 3. The predicted impact of various rules and consistency checks on proportions of tests held for manual review and verification were evaluated using downloaded patient test results from the laboratory information system. A description of consistency check rules and calculations are summarized in Table 4 and the notes back to MLTs for each are summarized in Table 5. Following implementation of the new AV system several new tools were implemented in order to allow continuous monitoring of the impact of the new system on error detection (Fig. 2) and in order to standardize evaluation of extreme values (Fig. 3A) and delta checks (Fig. 3B) to compliment the automated comments to MLTs concerning consistency checks and HIL failures. The impact of the new AV system compared to the original one relative to time spent by MLTs for review and release of held tests are summarized in Table 6.

**Table 1:** Summary of activities by phase of the AV project.

**Phase**	**Description**	**Activities**	**Relative start time (Duration)**
**Define**	Most elements of project planning were carried out during this phase of the work.	• Identify Sponsor(s) • Draft Project Charter • Draft & finalize Schedule • Select/Prepare project team • Construct top level process map • Construct top level SIPOC^a^ diagram • Identify metrics • Finalize Project Charter	Week 1 (2 weeks)
**Measure**	This phase involved defining, evaluating, and implementing a system for measuring the AV process.	• Construct process maps for AV • Select metrics • Develop/Implement monitoring system • Begin data collection • Evaluate the measurement system	Week 3 (10 weeks)
**Analyze**	This phase involved developing AV benchmarks and targets; and analyzing and interpreting data to inform decisions on improvements.	• AV Value Stream Analysis • Determine AV benchmark • Perform AV variance analysis • Perform Root cause analysis • Analyze requirements and process drivers summarize analyses	Week 13 (2 weeks)
**Improve**	This phase involved development and implementation of new AV process.	• Prioritize improvement opportunities • Design new AV process • FMEA^b^ for new AV process • Implement new AV process • Examine early data from new process and optimize parameters • Feedback meetings with MLTs	Week 15 (8 weeks)
**Control**	This phase involved verification of improvements and development of a control plan to maintain the new AV process.	• Confirm/validate new AV process • Develop and implement SOPs^c^ and monitoring plan • Assign a monitor • Approve of deliverables • Project closeout and review	Week 23 (8 weeks)

a SIPOC (Suppliers, Inputs, Process, Outputs, Customers).

b FMEA (Failure Modes and Effects Analysis).

c SOP (Standard Operating Procedures).

**Table 2:** Summary of metrics and targets for the new AV system.

***Performance metrics***	**Definition/units**	**Baseline**	**Benchmark or target**
***Samples Held***	Proportion of samples analyzed per week.	HSC: 0.398 ± 0.037 (*n* = 6)	< 0.10
		WMH: 0.650 ± 0.014(*n* = 6)	
		SCH: 0.604 ± 0.036(*n* = 6)	
***Tests Held***	Proportion of all tests analyzed per week.	HSC: 0.225 ± 0.009 (*n* = 6)	< 0.10
		WMH: 0.209 ± 0.009(*n* = 6)	
		SCH: 0.223 ± 0.012(*n* = 6)	
***Potassium Tests Held by HIL Flags***	Proportion of all potassium tests per week.	3.7%	< 2.5%
***Potassium Tests Held by Delta Check***	Proportion of all potassium tests per week.	3.7%	< 2.5%
***Potassium Tests Held by High/Low***^a^	Proportion of all potassium tests per week.	12.8%	< 1%
***Potassium Tests Held for Consistency Check***	Proportion of all potassium tests per week.	1.6%	< 2.5%
***Process Time***	Median time (minutes) from placement on track to result release to electronic medical record per week.	HSC: 41.3 ± 1.00^b^ (*n* = 6)	≤ baseline
		WMH: 32.8 ± 1.2^b^ (*n* = 7)	
***Total Time for Result Verification***	Weekly labor time associated with review of tests held for manual review (calculated from the “Test Manual Verification Time” and average number of samples held per week).	16,785 ± 5461 s	> 50% reduction
***Test Manual Verification Time***	Average time (seconds) spent reviewing held samples.	7.1 ± 4.0 (Mean ± SD)	≥ baseline

a Outside of upper (High) and lower (Low) limit of normal.

b Based on time specimen on automated track system at HSC, but from time of receipt in the laboratory at WMH. Expressed as average weekly median and standard deviation.

**Table 3:** Pre-existing and predicted (for new AV process) proportion of tests held for manual review for AV components and consistency check rules. Frequency of tests being held and predicted rates are based on HSC data. Hold rates were determined by analyzing total tests held by criteria over a two week period from March 27 to April 10, 2017 and involving 80,876 tests from HSC. Similar data was also used to predict future AV hold rates for the new rules.

**Result hold rules**	**Proposed**	**Test hold rate**	c**Predicted rate**
**Delta check**	Use 0.025 and 0.975 percentiles to set limits.^a^^,^^b^	0.0128	< 0.005
**Extreme values**	Use 0.0025 and 0.9975 percentile estimates versus reference ranges	0.1962	< 0.005
**HIL flags**	Hold only results that require action only.	0.0102	< 0.0025
**Critical values**	Critical values will be maintained without modification	0.0073	< 0.01
**Consistency checks**			
**Anion gap**	Less than 4 or greater than 20	0.0073	No hold
**BUN/creatinine ratio**		New	No hold
**A/P ratio**	New absurdity rule	New	< 0.0001
**Transaminase rule**^c^	New rule based on 0.0025–0.0075 percentile	New	< 0.005
**DB/TB Ratio**^c^	New absurdity rule	New	< 0.0001
**Both TSH and fT4 > upper reference limit**	New rule for rare and unusual observations (secondary or tertiary hyperthyroidism)	New	< 0.0005
**Both TSH and fT4 < lower reference limit**	New rule for rare and unusual observations (secondary or tertiary hypothyroidism)	New	< 0.0005
**All indices (H, I, L) of ≥ 1+**	New rule for poor sample quality (vendor)	New	< 0.0001
**hemolysis is ≥ 2+ and lipemia flag is ≥ 1+**	New rule for poor sample quality (vendor)	New	< 0.0001
**hemolysis is 4+ and lipemia flag is ≥ 1+**	New rule for poor sample quality (vendor)	New	< 0.0001
**Ictchk1 = Total bilirubin - "I-index as concentration"**	New rule for interference in bilirubin assay	New	< 0.0001
**Sodium < 131, Chloride < 105, Potassium < 4.1 (Reflex Glucose > 20** **mmol/L)**	New rule to detect interference by dextrose solution	New	~ 0.001
**Sodium < 132, Chloride < 103, Potassium < 4.1. Glucose > 18**	New rule to detect interference by dextrose solution	New	< 0.0005
**Sodium < 126, Chloride < 105, Potassium < 3.8**	New rule to detect interference by dextrose solution	New	< 0.0005
**Potassium > 7 and (Calcium < 2, or ALP < 50, or Magnesium < 0.5)**	Existing rule to detect EDTA interference.	0.0002	< 0.0005
**HDL > Chol**	New absurdity rule	New	< 0.0001
**Anion Gap < 1**		New	< 0.0001

a Park et al. [2].

b Lee et al. [3].

c New rules with no occurrence in the data set were assigned a predicted frequency < 0.0001.

**Table 4:** Consistency check calculations and rule definition.

**Number**	**Test**	**Calculation/Logic**
1	BUN/Creat ratio	BUN/Creat ratio = Urea/(Creatinine/1000) (Information rule only)
2	AG	AG = Sodium - Chloride - Total CO2 (Information rule only)
3	DB/TB ratio	DB/TB ratio = Direct Bilirubin/Total Bilirubin (> 1 will flag)
4	A/P ratio	A/P ratio = Albumin/Total Protein (beyond 0.25 or 1 will flag)
5	Transam ratio	Transam ratio = ALT/AST (beyond 0.25 or 4 will flag)
6	T4 high rule	Both TSH and fT4 greater than upper reference limit
7	T4 low rule	Both TSH and fT4 less than lower reference limit
8	HIL all positive	All indices (H, I, L) of one plus or greater.
9	H-L flag 1	hemolysis is ≥ 2+ and lipemia flag is ≥ 1+
10	H-L flag 2	hemolysis is 4+ and lipemia flag is ≥ 1+
11	Icterror	Ictchk1 = Total bilirubin - "I index as concentration" (> 65 will flag)
12	ContSamp1	Sodium < 130, Chloride < 100, Potassium > 5.5
13	ContSamp2	Sodium < 131, Chloride < 105, Potassium < 4.1 (Reflex glucose > 20 mmol/L)
14	ContSamp3	Sodium < 132, Chloride < 103, Potassium < 4.1. Glucose > 18
15	ContSamp4	Sodium < 126, Chloride < 105, Potassium < 3.8
16	Ivglu	Sodium < 136,Chloride < 98, Potassium > 5.5, Glucose > 6.0
17	IVglu2	Negative delta for sodium and chloride and positive delta for glucose and potassium
18	IVsalinecont1	Sodium > 160, Chloride > 110, Potassium < 3.5, glucose < 3.3
19	Ivsalinecont2	Positive delta for Sodium and Chloride and negative delta for glucose and potassium
20	EDTA Check	Potassium > 7 and (Calcium < 2, or ALP < 50, or Magnesium < 0.5)
21	Tchol-HDL	Tchol-HDL = HDL/Chol (>0.75 will flag)
22	Delay Check	Glucose < 2.21,Potassium > 6, hemolysis index < 50 or negative
23	Fibrin Check	Sodium< 136, Potassium < 3.5, Calcium < 2.1, Glucose < 3.9 (and negative deltas)
24	Mixup1	Delta calculation (((Current Creat -Past Creat)/Past Creat)/days)*100% (beyond − 50% or + 50% will flag)
25	Mixup2	Delta calculation ((Current Creat -Past Creat)/Past Creat)*100% (beyond 50% will flag)
26	AGLow	Anion Gap < 1

**Table 5:** Notes to MLTs for consistency checks and HIL flags.

Comment code	Note to MLT
AGRule	Repeat electrolyte measurements unless patient previously had similarly abnormal anion gap. If not confirmed investigate for analytical errors affecting electrolytes. Unless sodium or albumin are low, very low anion gaps (< 1) may be caused by analytical error.
A-Prule	Repeat albumin and total protein on a different instrument. Perform QC check. Contact physician/unit to discuss if required.
BUN-Crule	Use when unusual urea or creatinine results. Repeat BUN and creatinine on a different instruments. Perform QC check. Contact physician/unit to discuss if required. Normal ratio 40–100; > 100 in prerenal failure; < 40 intrinsic renal disease.
ContSamp	Suppress all results, call ward and determine if sample collected from line. Contamination Risk!!!
DB-TBRule	Repeat Direct and Total bilirubin on a different instrument. Perform QC check. Contact physician/unit to discuss if required.
Delaychk	Possible specimen delay error!!!. Examine collection time and investigate.
Delt	Determine if result is expected. Contact physician/unit to discuss if necessary. If not expected, recommend recollection.
EDTAchk	Examine calcium, or magnesium, or ALP results for potential EDTA interference. (All will be very low!)
Fibrinchk	Possible Fibrin error!!! Especially if accompanied by negative deltas. Inspect sample, re-centrifuge and reanalyze.
HDLCHchk	Repeat HDL and total cholesterol on a different instrument. Perform QC check. Contact physician/unit to discuss if required.
Hem4+	Inspect sample for gross hemolysis. If confirmed, report no result and recommend specimen recollection.
HILallfail	Possible indice error. Please visually inspect sample and verify all results if there are not sample quality concerns
HLflag1	Inspect sample for lipemia. Verify results if there are no sample quality concerns.
HLflag2	Inspect sample for lipemia and confirm sample has been centrifuged. Reject if sample has been centrifuged!
Ict	Inspect sample for icterus. If confirmed, report no result for test.
Icterror	Possible paraprotein interference in bilirubin assay! Obtain the I-index value, repeat total bilirubin on a different analyzer, measure direct bilirubin, and correlate with SPE results. If SPE has monoclonal protein and "Icterror" confirmed, do not report bilirubin results - report possible paraprotein interference.
Lip	Inspect Sample. Ultracentrifuge and rerun all ordered chemistries (excluding lipids).
T4Rule	Repeat fT4 and TSH on a different instrument. Perform QC check. Contact physician to discuss if required.
TransRule	Repeat AST and ALT on a different instrument. Perform QC check. Contact physician/unit to discuss if required.
UHRammonia	Compare sample age with analysis time. Samples should be promptly analyzed < 1 h of collection. Consult specimen test stability table.
Mixchk	Investigate specimen for mix-up. Correlate with changes in other tests and rule out renal failure and dialysis patients.
UCREL	Note very low urine creatinine! Correlate with serum creatinine and other tests.

**Table 6:** Average time for release of samples by MLTs during manual verification. Manual result verification time studies were conducted at HSC site by an observer using a stop watch and timing technologists as they went about manual review activities. Verification time was determined from point of first appearance of result profile to release of results to the electronic record. Appearance of critical results were sporadic but these time periods were removed as they were very variable in length, proportionately more common during the post-improvement stage, and tended to skew average time per sample verified.

**MLT**		**Number of samples**	**Seconds per sample**		**Number of samples**	**Seconds per sample**
**1-SB**	**Pre-improvement**	72	6.57	**Post-improvement**		
**2-R**		123	7.83			
**3-W**		213	6.01			
**4-A**		100	16.58		86	12.03
**5-DC**		204	4.90		11	18.00
**6-AM**		42	5.00		45	31.91
**7-K**		109	5.10		58	15.76
**8-Cas**		100	5.05		13	19.62
**9-L**					7	29.29
**10-N**					23	20.43
**All**			7.13 ± 3.95			21.01 ± 7.15*

* Statistically significant based on *p* < 0.001 by Student *T* test for independent samples.
