# Supplementary material for: Strategy for 90% autoverification of clinical chemistry and immunoassay test results using six sigma process improvement
Source: Data Brief. 2018 May 3;18:1740–9. doi: 10.1016/j.dib.2018.04.080 (PMC5998219; doi:10.1016/j.dib.2018.04.080)
Supplement: Supplementary file 1 — Supplementary material [file mmc1.docx]

**Conflict of interest**

There are no conflicts of interest by any author of this manuscript.
